# A multi-stage drop-the-losers design for multi-arm clinical trials

**DOI:** 10.1177/0962280214550759

**Published:** 2016-09-30

**Authors:** James Wason, Nigel Stallard, Jack Bowden, Christopher Jennison

**Affiliations:** 1 MRC Biostatistics Unit, Cambridge, UK; 2 Warwick Medical School, University of Warwick, Coventry, UK; 3 Department of Mathematical Sciences, University of Bath, Bath, UK

**Keywords:** clinical trial design, delay, group-sequential designs, interim analysis, multi-arm multi-stage designs, multiple testing

## Abstract

Multi-arm multi-stage trials can improve the efficiency of the drug development process when multiple new treatments are available for testing. A group-sequential approach can be used in order to design multi-arm multi-stage trials, using an extension to Dunnett’s multiple-testing procedure. The actual sample size used in such a trial is a random variable that has high variability. This can cause problems when applying for funding as the cost will also be generally highly variable. This motivates a type of design that provides the efficiency advantages of a group-sequential multi-arm multi-stage design, but has a fixed sample size. One such design is the two-stage drop-the-losers design, in which a number of experimental treatments, and a control treatment, are assessed at a prescheduled interim analysis. The best-performing experimental treatment and the control treatment then continue to a second stage. In this paper, we discuss extending this design to have more than two stages, which is shown to considerably reduce the sample size required. We also compare the resulting sample size requirements to the sample size distribution of analogous group-sequential multi-arm multi-stage designs. The sample size required for a multi-stage drop-the-losers design is usually higher than, but close to, the median sample size of a group-sequential multi-arm multi-stage trial. In many practical scenarios, the disadvantage of a slight loss in average efficiency would be overcome by the huge advantage of a fixed sample size. We assess the impact of delay between recruitment and assessment as well as unknown variance on the drop-the-losers designs.

## 1 Introduction

Testing multiple experimental treatments against a control treatment in the same trial provides several advantages over doing so in separate trials. The main advantage is a reduced sample size due to a shared control group being used instead of a separate control group for each treatment. Other advantages include that direct comparisons can be made between experimental treatments and that it is administratively easier to apply for and run one multi-arm clinical trial compared to several traditional trials.^1^ Multi-arm multi-stage (MAMS) clinical trials include interim analyses so that experimental treatments can be dropped if they are ineffective; also, if desired, the trial can be designed so that it allows early stopping for efficacy if an effective experimental treatment is found. Two current MAMS trials that are ongoing are the MRC STAMPEDE trial,^1^ and the TelmisArtan and InsuLin Resistance in HIV (TAILoR) trial (the design of which is discussed in Magirr, Jaki and Whitehead^2^).

Magirr et al.^2^ extend Dunnett’s multiple-testing procedure^3^ to multiple stages, which we refer to as the group-sequential MAMS design. In this design, futility and efficacy boundaries are prespecified for each stage of the trial. At each interim analysis, statistics comparing each experimental treatment to the control treatment are calculated and compared to these boundaries. If a statistic is below the futility boundary, then the respective experimental arm is dropped from the trial. If a statistic is above the efficacy threshold, the trial is stopped with that experimental treatment recommended. Boundaries would generally be required to control the frequentist operating characteristics of the trial. Since there are infinitely many boundaries that do so, a specific boundary can be chosen to minimise the expected number of recruited patients at some treatment effect,^4^ or by using some boundary function such as those of Pocock,^5^ O’Brien and Fleming,^6^ or Whitehead and Stratton.^7^

The group-sequential MAMS design is efficient in terms of the expected sample size recruited, but has the practical problem that the sample size used is a random variable. This makes planning a trial more difficult than when the sample size is known in advance. An academic investigator applying for funding to conduct a MAMS trial will find that traditional funding mechanisms lack the required flexibility to account for a random sample size.^8^ Generally, they would have to apply for the maximum amount that could potentially be used, with the consequence that such trials appear highly expensive to fund. There are also several other logistical issues to consider, such as employing trial staff to work on a trial with a random duration.

An alternative type of MAMS trial is one in which a fixed number of treatments is dropped at each interim analysis. Stallard and Friede^9^ propose a group-sequential design where a set number of treatments is dropped at each interim analysis, and the trial stops if the best-performing test statistic is above a predefined efficacy threshold or below a predefined futility threshold. The stopping boundaries are set assuming the maximum test statistic is the sum of the maximum independent increments in the test statistic at each stage, which is generally not true and leads to conservative operating characteristics. A special case of Stallard and Friede’s design is the well-studied two-stage drop-the-losers design,^10,11^ in which one interim analysis is conducted, and only the top-performing experimental treatment and a control treatment proceed to the second stage. In Thall et al.,^10^ the chosen experimental treatment must be sufficiently effective to continue to the second stage. More flexible two-stage designs have been proposed by several authors, including Bretz et al.^12^ and Schmidli et al.^13^ These designs used closed testing procedures and/or combination tests to control the probability of making a type-I error whilst allowing many modifications to be made at the interim. In the case of multiple experimental arms, there is more scope for improved efficiency by including additional interim analyses, at least for group-sequential MAMS designs.^2,4^

In this paper, we extend the two-stage drop-the-losers design to more than two stages and derive formulae for the frequentist operating characteristics of the design. The resulting design has the advantage of a fixed sample size by maintaining a prespecified schedule of when treatments are dropped. That is, at each interim analysis, a fixed number of treatments are dropped. Note that this could be thought of as subdividing the first stage of a two-stage drop-the-losers trial to allow multiple stages of selection. We show that when there are several treatments, allowing an additional stage of selection noticeably decreases the sample size required for a given power, compared to the two-stage design. We also compare the multi-stage drop-the-losers design to the Dunnett-type MAMS design.

## 2 Notation

We assume that the trial is to have *J* stages, that is, *J* − 1 interim analyses and a final analysis, and starts with *K* experimental treatments and a control treatment. Let k∈{0,1,…,K} index the treatment (*k* = 0 represents the control treatment). Cumulative up to the end of the *j*th stage of the trial, a total of *n_j_* patients have been recruited to each remaining treatment. The number of treatments to be dropped at each stage (i.e. values of *n_j_*) are prespecified, and in particular do not depend on the results of the trial. The *i*th patient allocated to treatment *k* has a treatment outcome, *X_ki_*, distributed as N(μk,σ2). The value of σ2 is assumed to be known.

For k∈{1,…,K}, define δk=μk-μ0. The null hypotheses to be tested are $H_{0}^{\left(k\right)}:\delta k\leq 0$. The global null hypothesis, *H_G_*, is defined as HG:δ1=δ2=…=δK=0. The known variance test statistic for treatment *k* at stage *j* is

(1) $$Z_{j}^{(k)}=(\frac{\sum_{i=1}^{nj}X\mathrm{ki}}{nj}-\frac{\sum_{i=1}^{nj}X0i}{nj})\sqrt{\frac{nj}{2\sigma 2}}$$

which has marginal distribution $N(\delta k\sqrt{\frac{nj}{2\sigma 2}},1)$.

The covariance between different test statistics can be shown to be

(2) $$\text{Cov}(Z_{j}^{\left(k\right)},Z_{l}^{\left(m\right)})=\{\sqrt{\frac{\min(nj,nl)}{\max(nj,nl)}}\text{if}\;k=m;\sqrt{\frac{1}{2}}\sqrt{\frac{\min(nj,nl)}{\max(nj,nl)}}\text{if}\;k\neq m$$

At each stage, a fixed and predetermined number of experimental treatments are dropped. Let *n*^(^*^j^*^)^ denote the number of experimental treatments continuing into stage *j*. For *J* stages, the design is denoted as a K:n(2):…:n(J-1):n(J) design, where K>n(2)>…>n(J-1)>n(J). Thus, at least one experimental treatment is dropped at each analysis. Although *n*^(^*^J^*^)^ can in principle be more than one, we henceforth only consider designs with *n*^(^*^J^*^) ^= 1, similar to a two-stage drop-the-losers design. The experimental treatments to be dropped are determined by ranking the $Z_{j}^{\left(k\right)}$ statistics of the remaining experimental treatments in order of magnitude, and removing the smallest (least promising) as prespecified by the design. The control treatment always remains in the trial. At the final analysis, one experimental treatment remains, and if its final test statistic is above a threshold, *c*, that treatment is recommended, and the respective null hypothesis rejected.

It is desirable that the design is chosen in order to control the family-wise type-I error rate (FWER). The FWER is the probability of rejecting at least one true null hypothesis, and strong control of the FWER at level *α* means that the FWER is ≤α for any configuration of true and false null hypotheses (i.e. for any values of δk, k=1,…,K). In Section 3, we demonstrate how to control the FWER at *δ*_1_ = *δ*_2_ =… = *δ_K_* = 0, and show in Section 4 that this strongly controls the FWER. As well as the FWER, it is also desirable to control the probability of selecting a genuinely good treatment, were it to exist. To formalise the latter quantity, we use the least favourable configuration (LFC) of Dunnett^3^ and consider the probability of recommending treatment 1 when *δ*_1_ = *δ*^(1)^ and *δ*_2_ = *δ*_3_ =… = *δ_K_* = *δ*^(0)^, where *δ*^(1)^ is a prespecified clinically relevant effect, and *δ*^(0)^ is some threshold below which a treatment is considered uninteresting. The configuration is called least favourable as it minimises the probability of recommending a treatment with effect greater than or equal to *δ*^(1)^ amongst all configurations where at least one treatment has a treatment effect of *δ*^(1)^ or higher and no treatment effects lie in the interval (*δ*^(0)^, *δ*^(1)^).^10^

## 3 Analytic operating characteristics

In this section, we provide analytical formulae for the probability of a particular treatment being recommended under a general vector of treatment effects. We also provide formulae for the probability of rejecting any null hypothesis when *H_G_* is true, and the probability to select the best treatment under the LFC. Although the formulae extend naturally to more than three stages, the expressions grow in length with the number of stages. For simplicity of exposition, we concentrate on the three-stage case, where *K* experimental treatments are included in the first stage, *L* < *K* in the second stage, and 1 in the third stage. This is denoted as the K:L:1 design.

### 3.1 Probability of a specific treatment being recommended

For subsequent development, it is useful to define a ranking of the experimental treatments in terms of how successful they are in the trial. We introduce random variables ψ=(ψ1,…,ψK), where *ψ_k_* is the ranking of treatment *k*. Each of the *ψ_k_*s takes a unique integer value between 1 and *K* with the following properties:

1. the treatment that reaches the final analysis has rank 1;
2. the treatment that is dropped at the first analysis with the lowest test statistic is given rank, *K*;
3. if treatment *k*_1_ reaches a later stage than treatment *k*_2_, then ψk1<ψk2, that is, treatment *k*_1_ has a higher ranking;
4. if treatments *k*_1_ and *k*_2_ are dropped at the same stage, and *k*_1_ has a higher test statistic at that stage, then ψk1<ψk2.

For instance, for a three-stage 4:2:1 design where treatment 3 reaches the final stage, treatment 2 is dropped at the second analysis, treatments 1 and 4 are dropped at the first analysis, and treatment 1 has the lowest test statistic at the first analysis, the realised value of *ψ* is (4, 2, 1, 3).

For *J* = 3, the probability of recommending treatment *k*, that is, rejecting $H_{0}^{\left(k\right)}$, given the mean vector δ=(δ1,δ2,…,δK) can be written in terms of *ψ* as

(3) $$P(\text{Reject}\;H_{0}^{\left(k\right)}|\delta )\;=\;P(\psi k=1,Z_{3}^{\left(k\right)}>c|\delta )$$

that is, the *k*th null hypothesis is rejected only if the *k*th experimental treatment reaches the final stage and its test statistic there is above the critical value *c*. Without loss of generality, consider the probability of recommending treatment 1. Let Ψ be the set of all possible realisation of *ψ*. Then the right-hand side of equation (4) becomes

(4) $$\sum_{\psi \in \Psi :\psi 1=1}P(Z_{3}^{\left(1\right)}>c,\psi |\delta )$$

We next show how each of the summands in equation (4) can be written as the tail probability of a multivariate normal distribution. The distribution of $Z=(Z_{1}^{\left(1\right)},Z_{2}^{\left(1\right)},Z_{3}^{\left(1\right)},\ldots ,Z_{1}^{\left(K\right)},Z_{2}^{\left(K\right)},Z_{3}^{\left(K\right)})$ is multivariate normal and we denote its mean by *m*(*δ*) and covariance by Σ, where these are defined by equations (1) and (2), respectively. Consider first the event that ψ1=1, ψ2=2,…,ψK=K, and $Z_{3}^{\left(1\right)}>c$. This event occurs if

$$Z_{1}^{\left(k\right)}>Z_{1}^{\left(L+1\right)}\;\text{for}\;\text{all}\;k=1,\ldots ,L$$

and

$$Z_{1}^{\left(L+1\right)}>Z_{1}^{\left(L+2\right)}>\ldots >Z_{1}^{\left(K\right)}$$

in order that treatments L+1,…,K are eliminated with the desired ordering after the first stage, and

$$Z_{2}^{\left(1\right)}>Z_{2}^{\left(2\right)}>\ldots >Z_{2}^{\left(L\right)}$$

so treatments 2,…,L are eliminated with the desired ordering after the second stage, and finally

$$Z_{3}^{\left(1\right)}>c$$

The specified event can be expressed in terms of conditions on differences between entries of *Z* plus the final condition $Z_{3}^{\left(1\right)}>c$. For example, the condition $Z_{1}^{\left(k\right)}>Z_{1}^{\left(L+1\right)}$ for all k=1,…,L is equivalent to the *L* inequalities

$$Z_{1}^{\left(1\right)}-Z_{1}^{\left(L+1\right)}>0,\ldots ,Z_{1}^{\left(L\right)}-Z_{1}^{\left(L+1\right)}>0$$

In all, there are K+L-2 inequalities involving pairs of elements of *Z* and one involving a single element of *Z*. This set of inequalities can be written in terms of a transformed variable *AZ* where *A* is a (K+L-1)×JK matrix and each of the first K+L-2 rows of *A* picks out the difference between two elements of *Z*, while the last row picks out $Z_{3}^{\left(1\right)}$. As an example, in the 4:2:1 design, the event $(\psi 1=1,\psi 2=2,\ldots \psi K=K,Z_{3}^{\left(1\right)}>c)$ has

A=(000000100-100100000-100000000100-1000000100-10000000001000000000)

and the requirements for the event to occur are

(AZ)i>0   for i=1,…,4   and   (AZ)5>c

Now, *AZ* is an affine transformation of a multivariate normal random variable, and so is normal with mean *Am*(*δ*) and covariance matrix AΣAT. Thus, the event $(\psi 1=1,\psi 2=2,\ldots \psi K=K,Z_{3}^{\left(1\right)}>c)$ can be expressed as a multivariate normal tail probability, which can be evaluated efficiently using the method of Genz and Bretz.^14^

Other terms in equation (4), in which the values of ψ2,…,ψK are different permutations of the indices 2,…,K, can be dealt with in a similar way. Computationally, one can simply permute the entries of the treatment effect vector *δ* in a suitable way so that the formulae for the case ψ1=1,ψ2=2,…,ψK=K can be applied and the matrix *A* and associated covariance matrix AΣAT remain unchanged.

The above approach extends directly to designs with more than three stages. For a K:n(2):n(3):…:n(J-1):1 design, at the end of stage j∈{1,…,J-1}, n(j)-1 conditions are imposed to ensure that the correct treatments are retained and the dropped treatments have the specified ordering. With one final condition to ensure that the *Z* statistic for the top-ranked treatment exceeds the critical value *c* at the final analysis, the total number of conditions is

$$1+(K-1)+\sum_{j=2}^{J-1}(n\left(j\right)-1)$$

so the matrix *A* has this number of rows and *JK* columns.

### 3.2 Probability of recommending any treatment under the global null hypothesis

When the global null hypothesis *H_G_* is true, each element of *m*(*δ*) is 0. By symmetry, the probability of observing each ordering *ψ* and a final *Z* statistic greater than *c* is the same. Thus, the probability of recommending any treatment under the global null hypothesis is

(5) $$K\;!\;\mathbb{P}(\psi 1=1,\psi 2=2,\ldots ,\psi K=K,Z_{J}^{\left(1\right)}>c|\delta =0)$$

and this needs the calculation of a single multivariate normal random variable, as described in Section 3.1.

### 3.3 Probability of recommending a specific treatment under the LFC

We assume the trial is to be powered to recommend treatment 1 at the LFC, where μ1-μ0=δ(1) and μk-μ0=δ(0) for k=2,…,K. Thus, the probability of recommending treatment 1 is

(6) $$(K-1)!\;\mathbb{P}(\psi 1=1,\psi 2=2,\ldots ,\psi K=K,Z_{J}^{\left(1\right)}>c|\delta 1=\delta \left(1\right),\delta 2=\delta \left(0\right),\ldots ,\delta K=\delta \left(0\right))$$

and this can be calculated as (K-1)! times the tail probability of a single multivariate normal random variable.

R code provided online (https://sites.google.com/site/jmswason) allows the user to find the values of *n* and *c* so that a design has required FWER and power.

## 4 Strong control of FWER

We can control the probability of recommending an ineffective treatment when the global null hypothesis *H_G_* is true by specifying the critical value *c* so that the probability (5) is equal to *α*. In the case of a group-sequential MAMS trial, controlling the error rate under *H_G_* has been shown to control the FWER in the strong sense.^2^ In this section, we prove that controlling the FWER at the global null hypothesis strongly controls the FWER for the multi-stage drop-the-losers design also.

We denote by *m_j_*, the fixed number of observations collected in stage *j* on each surviving treatment and on the control arm. At the end of stage *j*, the cumulative sample size on each remaining treatment and the control arm is nj=m1+…+mj. Without loss of generality, we assume just one treatment is eliminated in each stage: the reason there is no loss of generality here is that if two or more treatments are to be eliminated, we can suppose that data-gathering stages with sample size *m_j_* = 0 take place between each elimination.

Initially the set of indices of all treatments is

I0={1,…,K}

and after a treatment has been eliminated at the end of stage *j*, we denote the set of indices of the *K* − *j* remaining treatments by *I_j_*.

Recall for k=1,…,K, we denote the observations on treatment *k* in stages 1 to *j* by *X_ki_*, i=1,…,nj, and denote the corresponding observations on the control arm by X0i,i=1,…,nj. For each k∈Ij-1, the difference between the sum of responses on treatment *k* and the control at the end of stage *j* is

$$Sj,k=\sum_{i=1}^{nj}(X\mathrm{ki}-X0i)$$

We define the terms *S_j_*_,_*_k_* for k∈Ij-1 since these are the statistics observed after gathering new data in stage *j*. The values Sj,k,k∈Ij-1, are used to select the treatment to be eliminated at the end of stage *j*, and the values Sj,k,k∈Ij, are then carried forward. The set IK-1 contains just one treatment index and after data are gathered on this treatment and control in stage *K*, this *S_j_*_,_*_K_* is used to decide whether or not the one treatment in IK-1 is superior to the control.

We first consider the general case where treatments 1 to *K* have treatment effects δ1,…,δK relative to the control treatment. For notational convenience, we set

S0,k=0,   k=1,…,K

With normally distributed responses of common variance σ2, we can describe the data gathering in stage j≥1 by writing

(7) $$Sj,k=Sj-1,k+mj\delta k+\varepsilon j,k\sqrt{mj\sigma 2}+\xi j\sqrt{mj\sigma 2}$$

where all the εj,k and *ξ_j_* are independent *N*(0, 1) random variables. Here, εj,k is associated with the responses on treatment *k* in stage *j*; *ξ_j_* is associated with responses on the common control arm in stage *j* and these terms introduce correlation into the sums Sj,k,k∈Ij-1.

After the data-gathering part of stage *j*, the treatment $k_{j}^{*}$ with the lowest *S_j_*_,_*_k_* for k∈Ij-1 is eliminated, leaving

$$Ij=Ij-1\backslash \{k_{j}^{*}\}$$

After the penultimate stage *K* − 1, one treatment, *k*_last_ say, remains in IK-1 and this treatment and the control are observed in the final stage, *K*. After stage *K*, the statistic including the final-stage data is SK,klast. If

SK,klast>c

H0:δklast≤0 is rejected in favour of δklast>0.

The trial is designed to have type-I error probability *α* when δ1=…=δK=0. We wish to show this also implies strong control of the FWER for testing the family of hypotheses $H_{0}^{\left(k\right)}:\delta k\leq 0$, k=1,…,K.

Consider two trials that have the same design but differ with respect to values of the treatment effects. In Trial 1, δ1=…=δK=0 and we use the notation described above. We define a parallel set of notation for Trial 2. We denote the treatment effects in Trial 2 by φl, l=1,…,K, and suppose some of the φl may be positive, and others negative or equal to zero. Let *L_j_* denote the set of indices of treatments still in the trial after stage *j* of Trial 2 and

Nj={l:l∈Lj   and   φl≤0}

so a type-I error will only occur if one of the hypotheses H0:φl≤0 for l∈Nj is eventually rejected. For j=1,…,K-1, let Tj,l,l∈Lj-1 be the analogues of Trial 1’s Sj,k,k∈Ij-1. For *j* = *K*, LK-1={llast},IK-1={klast} and TK,llast is the analogue of SK,klast.

With

T0,l=0,   l=1,…,K

we can write for each j≥1

(8) $$Tj,l=Tj-1,l+mj\varphi l+\eta j,l\sqrt{mj\sigma 2}+\xi j\sqrt{mj\sigma 2}$$

where the *η_j_*_,_*_l_* and *ξ_j_* are independent *N*(0, 1) random variables.

After the data-gathering part of stage *j*, the treatment $l_{j}^{*}$ with the lowest *T_j_*_,_*_l_* for l∈Lj-1 is eliminated, leaving

$$Lj=Lj-1\backslash \{l_{j}^{*}\}$$

After the penultimate stage *K* − 1, only one treatment, *l*_last_ say, remains. This is observed in stage *K* and if

TK,llast>c

H0:φllast≤0 is rejected in favour of φllast>0.

We shall establish the desired FWER property by a coupling argument, which assumes the terms *ξ_j_* in equations (7) and (8) are equal and which reuses values *η_j_*_,_*_l_* in equation (8) as values for some of the εj,k in equation (7). It is straightforward to see that the model for Trial 1 given by equation (7) and the model for Trial 2 given by equation (8) follow the correct distributional assumptions. The type-I error rate for Trial 1 is *α*, by construction. Thus, if we can demonstrate that a type-I error is made in Trial 1 whenever a type-I error is made in Trial 2, it follows that Trial 2 has the smaller type-I error probability – and so this must be no greater than *α*.

A key step in the coupling argument is to define the relationship between treatments k∈Ij-1 and l∈Lj-1, which specifies how values *η_j_*_,_*_l_* in equation (8) are to be used as values for the εj,k in equation (7). Define

N0={l:φl≤0}

and, as noted previously,

Nj={l:l∈Lj   and   φl≤0},   for j=1,…,K-1

For *j* = 0, define

π0(l)=l,   for eachl∈N0

In applying equation (8) for *j* = 1, generate independent random variables ξ1~N(0,1) and η1,l~N(0,1), l∈L0. Then, in applying equation (7) for *j* = 1, use the same value *ξ*_1_ as in equation (8), set

ε1,π0(l)=η1,l   for each l∈N0

and generate the remaining ε_1,_*_k_* values as additional independent *N*(0, 1) variates. It follows that

(9) T1,l≤S1,π0(l)   for each l∈N0

Our aim is to define injective functions *π_j_* from *N_j_* to *I_j_* at the end of each stage j=1,…,K-1, such that

(10) Tj,l≤Sj,πj(l)   for each l∈Nj

Intuitively, this means that for each treatment arm in Trial 2 that has a treatment effect less than or equal to zero, and so would produce a type-I error if the associated null hypothesis were rejected, there is a treatment arm in Trial 1 which has a treatment effect of zero and more positive current data – and so this should be more inclined to lead to a type-I error. Finally, after stage *K*, we have the control and just one treatment, *k*_last_ in Trial 1 and *l*_last_ in Trial 2 and final statistics SK,klast and TK,llast.

Assuming we can define the desired functions *π_j_*, there are two possibilities at the end of the trial when stage *j* = *K* is completed. The first possibility is that, on entering stage *K*, the set NK-1 is empty and a type-I error cannot be made in Trial 2. The second is that NK-1 is nonempty and contains a single element, so φllast≤0 and πK-1(llast)=klast (the only element of IK-1): before the final-stage data are seen

TK-1,llast≤SK-1,klast

then with the (coupled) final-stage data

TK,llast≤SK,klast

A type-I error in Trial 2 requires TK,llast>c and this can only occur if

SK,klast>c

in which case a type-I error is also made in Trial 1. This establishes the desired property that a type-I error is made in Trial 1 whenever a type-I error is made in Trial 2 and the FWER result follows.

It remains to show that injective functions *π_j_* from *N_j_* to *I_j_*, j=1,…,K-1, can be defined with the required property as expressed in equation (10). For the case *j* = 1, we know that equation (9) holds before a treatment is eliminated at the end of stage 1 and we need to define a function *π*_1_ from *N*_1_ to *I*_1_ satisfying equation (10) with *j* = 1, after the first treatment has been eliminated. The eliminated treatments are $k_{1}^{*}$ in Trial 1 and $l_{1}^{*}$ in Trial 2, where

(11) $$S1,k_{1}^{*}\leq S1,k\;\text{for}\;k\in I0,k\neq k_{1}^{*}$$

and

$$T1,l_{1}^{*}\leq T1,l\;\text{for}\;l\in L0,l\neq l_{1}^{*}$$

In defining *π*_1_ from *N*_1_ to *I*_1_, we need to consider values $l\in N1=N0\backslash \{l_{1}^{*}\}$. For each value l∈N1 with $\pi 0(l)\neq k_{1}^{*}$, we set

$$\pi 1(l)=\pi 0(l)\in I1=I0\backslash \{k_{1}^{*}\}$$

It follows from equation (9) that T1,l≤S1,π1(l) for these values of *l*. Now suppose there is a value $\overset{\sim }{l}\in N1$ for which $\pi 0(\overset{\sim }{l})=k_{1}^{*}$ and thus $\pi 0(\overset{\sim }{l})\notin I1=I0\backslash \{k_{1}^{*}\}$. In this case, we can set $\pi 1(\overset{\sim }{l})$ to be any index in *I*_1_, which is not already defined as π1(l) for some other l∈N1 (since *I*_1_ has at least as many elements as *N*_1_, there will be at least one option to choose here). The resulting *π*_1_ has the injective property. Now, by equations (9) and (11)

$$T1,\overset{\sim }{l}\leq S1,\pi 0(\overset{\sim }{l})=S1,k_{1}^{*}\leq S1,\pi 1(\overset{\sim }{l})$$

so equation (10) is satisfied for *j* = 1 and $l=\overset{\sim }{l}$. This completes the definition of *π*_1_.

The construction of functions *π_j_* for j=2,…,K-1 and proof of their properties continues by induction. For a general *j*, we apply equations (7) and (8) using the same *ξ_j_* in both cases and with

εj,πj-1(l)=ηj,l   for each l∈Nj-1

With property (10) for *j* − 1, we have

Tj-1,l≤Sj-1,πj-1(l)   for each l∈Nj-1

and because of the common values of εj,πj-1(l) and *η_j_*_,_*_l_* and the common *ξ_j_* arising in equations (7) and (8), this ensures that

Tj,l≤Sj,πj-1(l)   for each l∈Nj-1

Thus, we can define *π_j_* by setting

πj(l)=πj-1(l)∈Ij

for each value l∈Nj with $\pi j-1(l)\neq k_{j}^{*}$. If there is a value $\overset{\sim }{l}\in Nj$ for which $\pi j-1(\overset{\sim }{l})=k_{j}^{*}$, we can set $\pi j(\overset{\sim }{l})$ to be any element of *I_j_* which is not already defined as πj(l) for some other l∈Nj. The same reasoning as in the case *j* = 1 shows that the resulting *π_j_* from *N_j_* to *I_j_* has the injective property and satisfies equation (10), which proves the inductive step.

As noted earlier, if φlast≤0, the inductive properties at stage *K* imply that before collecting the final-stage data, we have πK-1(llast)=klast and

TK-1,llast≤SK-1,klast

then with the (coupled) final-stage data,

TK,llast≤SK,klast

A type-I error in Trial 2 requires TK,llast>c and this can only occur if

SK,klast>c

in which case a type-I error is also made in Trial 1, as required.

## 5 Results

### 5.1 Motivating trial

As a case study for the results in this paper, we consider the currently ongoing TAILoR trial, the design of which is discussed in Magirr et al.^2^ This trial was originally designed to test four different doses of Telmisartan. Telmisartan is thought to reduce insulin resistance in HIV-positive individuals on combination antiretroviral therapy. The primary end point was reduction in insulin resistance in the telmisartan-treated groups in comparison with the control group as measured by homeostatic model assessment – insulin resistance (HOMA-IR) at 24 weeks. A group-sequential MAMS design was used to avoid assumptions regarding monotonicity of dose–response relationship, which were thought to be invalid based on a previous trial of the treatment in a different indication.

The trial design controls the FWER at 0.05 with 90% power under the LFC with δ(1)=0.545,δ(0)=0.178,σ2=1. The value of *δ*^(1)^ was chosen so that the probability of a patient allocated to a treatment with treatment effect *δ*^(1)^ having a better treatment response than a patient, given the control treatment was 0.65. The value of *δ*^(0)^ was chosen to make the corresponding probability 0.55.

### 5.2 Comparison of two- and three-stage drop-the-losers designs

We first show that extending the drop-the-losers design beyond two stages can be worthwhile. For (α,1-β,δ(1),δ(0))=(0.05,0.9,0.545,0.178), and selected values of *K*, we used equations (5) and (6) to find the required sample size of the one-stage design (with no interim analysis), a two-stage drop-the-losers design and a three-stage drop-the-losers design. For each multi-stage design, a value *n* is specified and *n* patients are assigned to each remaining treatment and the control in each stage. For each three-stage design, the number of treatments proceeding to stage 2 was chosen to give the lowest total sample size.

Table 1 shows the required total sample size for each type of design when there are *K* = 3, 4, 6 and 8 experimental treatments (recall that the full sample size is always used, so there is no dependence of sample size on the actual treatment effects). The table also shows the percentage reduction in sample size when the number of stages is increased from 1 to 2 and from 2 to 3. The benefits gained by including a third-stage increase with the number of treatments. It is likely that at least *K* = 4 experimental treatments are necessary before the additional administrative burden of a third stage would be deemed worthwhile. For *K* as large as 6 or 8, the reduction in sample size in going from 1 to 2 stages is similar to that gained in moving from 2 to 3 stages, so if a first interim analysis is regarded as cost effective, then a second interim analysis should also be worthwhile.

**Table 1.** Sample sizes required for a one-stage design and two-stage and three-stage drop-the-losers designs with *α* = 0.05, *β* = 0.1, *δ*^(1) ^= 0.545 and *δ*^(0) ^= 0.178.

	Total sample size required for 90% power			Percentage reduction in sample size	
*K*	*J* = 1	*J* = 2	*J* = 3	*J* = 1 to *J* = 2	*J* = 2 to *J* = 3
3	312	282	270	9.6	4.2
4	420	364	330	13.3	9.3
6	637	531	455	16.6	14.3
8	864	715	585	17.2	18.2

*Note*: For each three-stage design, the number of treatments proceeding to stage 2 is chosen to give the lowest total sample size: in the notation of Section 2, these designs are 3:2:1 for *K* = 3, 4:2:1 for *K* = 4, 6:3:1 for *K* = 6 and 8:3:1 for *K* = 8.

### 5.3 Comparison of three-stage group-sequential MAMS and drop-the-losers designs

**Figure 1.**
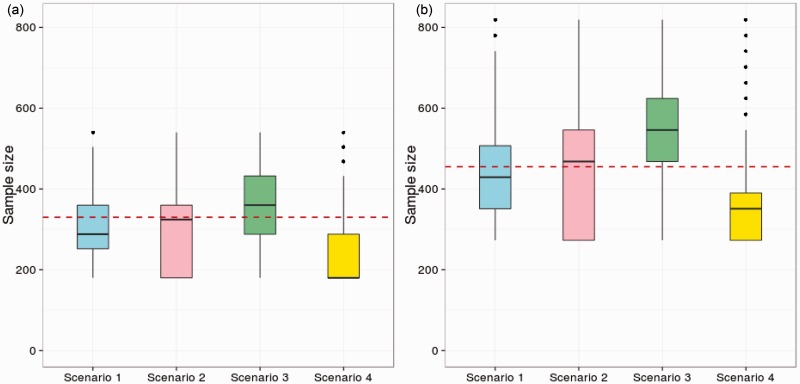
Sample size distribution for three-stage group-sequential MAMS designs with *K* = 4 and *K* = 6 and four vectors of treatment effects. Scenario 1 – the global null hypothesis (*H_G_*); scenario 2 – the LFC; Scenario 3 – all experimental treatments have uninteresting treatment effect *δ*^(0)^; Scenario 4 – all experimental treatments have effect -δ(0). The dashed red line gives the required sample size for the three-stage drop-the-losers design with the same parameters used: *α* = 0.05, *β* = 0.1, *δ*^(1) ^= 0.545, *δ*^(0) ^= 0.178.

We now compare sample size properties of drop-the-losers designs with those of group-sequential MAMS designs when design parameters are specified as in the previous section. The group-sequential MAMS designs have three analyses and use the triangular test boundaries of Whitehead and Stratton,^7^ which are known to give good expected sample size properties.^4^ Figure 1 shows boxplots of the sample size distribution (using 250,000 replicates) for the three-stage group-sequential MAMS designs with *K* = 4 and *K* = 6 experimental arms under four scenarios: (1) under *H_G_*; (2) under the LFC; (3) when *δ*_1_ = *δ*_2_ =… = *δ_K_* = *δ*^(0)^ and (4) when *δ*_1_ = *δ*_2_ =… = *δ_K_* = −*δ*^(0)^. The solid black line in each boxplot represents the median sample size. The dashed line for each *K* represents the fixed sample size of the most efficient three-stage drop-the-losers designs (4:2:1 for *K* = 4 and 6:3:1 for *K* = 6).

Although the group-sequential MAMS designs with triangular test boundaries are known to have low expected sample sizes, Figure 1 shows that the sample size distribution is highly variable and depends strongly on the configuration of treatment effects. If we take the median sample size of the group-sequential MAMS design as a point of comparison, we see the sample size for the drop-the-losers design is higher under *H_G_* (Scenario 1), almost equal under the LFC (Scenario 2) and lower when all treatment effects are equal to *δ*^(0)^ (Scenario 3). These results are generally encouraging for the drop-the-losers design and show that the constraint of a fixed total sample size can be met without sacrificing much efficiency in terms of average numbers of patients recruited.

The performance of the drop-the-losers design is poorest in Scenario 4 where all the treatment effects are negative and the MAMS designs are likely to stop the whole trial early for futility. Results for this scenario indicate the desirability of adding a futility rule to the drop-the-losers design: although some variation in total sample size would be introduced, ethical considerations argue against continued use of treatments which are proving ineffective. One might, for example, specify a minimum requirement for treatments to meet at each stage and allow fewer than the specified number to continue when some treatments fail to meet this requirement – or stop the trial completely if no treatment satisfies the requirement. If a rule of this type was superimposed on the drop-the-losers design with no other changes to sample numbers or the final critical value, *c*, the type-I error rate would simply be reduced. Alternatively, the calculations of Section 3.1 could be extended to include this form of futility rule and the design parameters adjusted to satisfy the type-I error rate requirement exactly.

## 6 Spacing of interim analyses when there is delay between recruitment and assessment of patients

In previous sections, we have assumed there is no delay between recruitment and assessment of patients. In reality, there will nearly always be some delay, and often it will be considerable. For example, in the TAILoR trial, the final end point is measured 24 weeks after treatment.

A delay between recruitment and assessment means that at the time of an interim analysis, there will be patients who have been recruited but not yet assessed, and thus contribute no information to that interim analysis. The efficiency of the trial, in terms of number of patients recruited, is then reduced as some patients will be recruited to arms that are dropped before their responses are measured. Also, with a delay in response there are fewer observations at each interim analysis and, thus, lower probabilities of selecting the best treatments. The potential loss of efficiency depends on the recruitment rate to the trial since this rate and the time at which the final end point is measured together determine the numbers of patients treated but not assessed at the interim analyses.

Hampson and Jennison^15^ have proposed ways of using partial information from patients who have been recruited but not assessed at the time of an interim analysis. If a short-term end point that is correlated with the final end point is available, fitting a joint model for both end points can increase the information for the final end point. When the final end point is the incidence of an event before a certain time, t* say, inference can be based on a Kaplan–Meier estimate of the probability of the event occurring before t*. In this case, the time-to-event data for all patients is used, with right censoring applying when the follow-up time is less than t* and the event has not yet occurred.

When there is a delay in response, the methodology described in Sections 3.1–3.3 can still be applied by conducting analyses at times when the required numbers of observations become available. We have explored the optimal spacing of analyses when there is a known delay. Since we have efficient computational methods for drop-the-losers designs, it is quite feasible to explore a wide variety of spacings. We report results for an example in which the primary end point is measured 24 weeks after recruitment, as in the TAILoR trial, and we consider recruitment rates of *m* = 1, 2 and 4 patients per week. The limiting case *m* = 0 is also included to represent the case of an immediate response.

We consider the 4:2:1 and 4:1 designs with, as before, *δ*^(1)^ = 0.545, *δ*^(0)^ = 0.178, *α* = 0.05 and 1-β=0.9. We have explored a grid of possible spacings for each design. For the 4:2:1 design, spacings are expressed in terms of parameters (1, *ω*_2_, *ω*_3_) defined as follows: if the initial group size of a design is *n* and the spacing is (1, *ω*_2_, *ω*_3_), the first interim analysis takes place after *n* patients have been recruited to each treatment arm, the second after a further *ω*_2_*n* patients have been recruited to each remaining arm and the last analysis occurs after recruiting and assessing a further *ω*_3_*n* patients on the remaining treatment and control arms. Thus, the total numbers recruited by analyses 1 and 2 are 5*n* and 5*n* + 3*ω*_2_*n*, respectively, but the numbers of observations seen at these analyses are lower since not all of these patients have been assessed. At the final analysis, all 5*n* + 3*ω*_2_*n* + 2*ω*_2_*n* patients have been assessed. We assume that once the decision has been made to drop an experimental arm, that decision cannot be reversed after seeing data from patients who were previously recruited but not assessed. For the 4:1 design, spacings are expressed in terms of parameters (1, *ω*_2_), where the first interim analysis takes place after *n* patients have been recruited to each treatment arm and an additional *ω*_2_*n* are recruited to the selected treatment and control in the second stage.

For each type of design, we searched over possible choices of *ω*_2_ and *ω*_3_ to find the design with the lowest total sample size. Table 2 shows the optimal values of *ω*_2_ and *ω*_3_ and the total sample size for two-stage and three-stage designs under specified values of *m*, the number of patients recruited per week. For comparison, the design that tests four experimental treatments without any interim analyses requires 420 patients in total. Table 2 shows the optimal spacing parameters and total sample size for both designs when the mean number of patients recruited per week, *m*, varies. Note that the design that tests four experimental treatments without any interim analyses requires 420 patients in total.

**Table 2.** Properties of 4:2:1 and 4:1 designs when there is a 24-week delay between recruitment and assessment.

	Optimal spacing		Max SS		Percentage reduction in SS	
*m*	*J* = 2	*J* = 3	*J* = 2	*J* = 3	*J* = 2	*J* = 3
0	(1, 0.9)	(1, 0.9, 0.8)	361	326	14.0	9.7
1	(1, 0.8)	(1, 0.9, 0.45)	377	344	10.2	8.8
2	(1, 0.5)	(1, 0.95, 0.2)	390	363	7.1	6.9
4	(1, 0.35)	(1, 0.75, 0.05)	422	405	−0.5	3.6

*Note*: A constant recruitment rate of *m* patients per week is assumed. Here, SS denotes sample size and *m* = 0 represents the limiting case when there is no delay in observing the response.

Table 2 shows that as the recruitment rate increases, there is a lower efficiency gain from including interim analyses. With a single interim analysis, the reduction in sample size of 14% in the case of immediate response falls to 7.1% when *m* = 2 and is lost completely for *m* = 4. The advantage of a three-stage design over a two-stage design also falls as *m* increases. Optimising the timing of the interim analyses is important here. As an example, with *m* = 2, a 4:2:1 design with equally spaced interim analyses, that is, (*ω*_2_, *ω*_3_) = (1, 1), needs a total of 390 patients, compared to the 363 patients for a design with the optimal spacing.

In view of these results, it is advisable to assess the likely impact of a delay in response on the efficiency of an adaptive design. Nevertheless, we have still seen that, for plausible combinations of recruitment rate and time to response, including either one or two interim analyses can reduce the sample size requirement compared to a design without interim analyses.

## 7 Discussion

MAMS designs are of great interest in practice, as their use means more new treatments can be tested with the same limited pool of patients. Much of the methodology about designing MAMS trials has focused on designs in which treatments are dropped early if their test statistics are below some prespecified futility boundary. This leads to variability in the number of treatments that will be in the trial at each stage, and therefore uncertainty in the total sample size required. This leads to uncertainties in applying for funding to conduct a MAMS trial, as well as other logistical issues such as staff employment. A design that does have a fixed sample size is the two-stage drop-the-losers design, where multiple experimental treatments are evaluated at an interim analysis, then the best-performing experimental treatment goes through to the second stage. We have investigated design issues in extending the drop-the-losers design to have more than two stages. If there are four or more treatments, we find that a third stage results in a considerable reduction in sample size. In addition, the fixed sample size compares well to the median sample size used in a group-sequential MAMS design. The design therefore retains many of the efficiency benefits of a MAMS design whilst also having a fixed sample size, which is very useful in practice. We have mainly considered the utility of adding a third stage, as each additional interim analysis increases the administrative burden of the trial. Adding a fourth stage provides a substantially lower additional efficiency advantage unless there are a lot of treatments being tested.

In this paper, we assumed a known variance of the normally distributed outcome. However, the method of quantile substitution, described in Section 3.8 of Jennison and Turnbull,^16^ can be used to change the final critical value so that the type-I error rate is controlled when the variance is estimated from the data. We carried out simulations that showed this method performs very well in practice (results not shown), similarly to the group-sequential^17^ and group-sequential MAMS cases.^4^

In practice, the requirement to drop a fixed number of treatments at each stage may be difficult to keep to. For example, if all treatments are performing poorly in comparison to control, then it may be unethical to continue with even the best performing treatment. Any changes to the design during the trial will affect the operating characteristics of the trial. However, dropping more treatments than planned will lead to a lower than nominal FWER rather than an inflation. If one wishes to keep more treatments in the trial than originally planned, then this will lead to an inflation in FWER. However, by modifying the final critical value suitably, this inflation can be reduced. The analytical formulae in this paper can be modified in order to calculate the required critical value if more sophisticated stopping rules are used.

An alternative design that controls the number of treatments passing each analysis but also allows early stopping of the trial for futility or efficacy is the design of Stallard and Friede.^9^ The multi-stage drop-the-losers design is somewhat less flexible than the Stallard and Friede design, but does have the advantage of having analytical formulae that provide exact operating characteristics of the design. The formulae for the Stallard and Friede design are conservative, especially when there are more than two stages. Of course simulation could be used to evaluate the operating characteristics exactly, but this makes it difficult to evaluate a large number of potential designs. We have shown that this is important in the case of delay between recruitment and assessment, where the spacing of the interim analyses becomes very important. The multi-stage drop-the-losers design can be evaluated extremely quickly, which allows the optimal interim analysis spacing to be found.

One worrying factor for the efficiency of adaptive trials in general, and the drop-the-losers design specifically, is delay between recruiting a patient and assessing their outcome. Such delay means that at a given interim analysis, there will be patients who are recruited but not yet assessed. These patients will not contribute to that interim analysis or to any subsequent analysis if the treatment they are on is dropped. We have investigated the effect of delay and show that drop-the-losers designs can still provide efficiency gains over a multi-arm design without interim analyses if the recruitment rate is below some level. This level will depend on the extent of delay and the total sample size of the trial. There are two factors that may go someway towards mitigating the impact of delay. Firstly, there may well be early outcomes that correlate well with the final outcome.^18^ For example, in the TAILoR trial, the final outcome is HOMA-IR at 24 weeks, but if earlier measurements could be made, these may well be highly informative for the 24 week end point. In that case, more patients could be included in the interim analysis. A second factor is that trial recruitment tends to start slowly and increase over time, perhaps as more centres are added to the trial. This means that a greater proportion of patients may be available for assessment at earlier interim analyses compared to the uniform recruitment case we considered here. Research into the effect of delay on group-sequential MAMS trials and strategies to account for it (extending the work of Hampson and Jennison^15^ to multi-arm trials) would be very useful.

This paper has considered design issues in multi-stage drop-the-losers trials. A drawback of adaptive designs in general is that estimation of relevant quantities, such as the mean treatment effect, after the trial is more complicated than in a traditional trial. For example, using the maximum likelihood estimate in two-stage trials will result in bias.^19,20,21^ The issue of estimation for multi-stage drop-the-losers trials is considered in Bowden and Glimm.^22^
